# Right ventricle to pulmonary artery coupling after transcatheter aortic valve implantation—Determinant factors and prognostic impact

**DOI:** 10.3389/fcvm.2023.1150039

**Published:** 2023-04-17

**Authors:** Catalina A. Parasca, Andreea Calin, Deniz Cadil, Anca Mateescu, Monica Rosca, Simona Beatrice Botezatu, Roxana Enache, Carmen Beladan, Carmen Ginghina, Dan Deleanu, Ovidiu Chioncel, Serban Bubenek-Turconi, Vlad A. Iliescu, Bogdan A. Popescu

**Affiliations:** ^1^ Cardiothoracic Department, University of Medicine and Pharmacy “Carol Davila”, Bucharest, Romania; ^2^ Cardiology and Cardiovascular Surgery Department, Emergency Institute for Cardiovascular Diseases “Prof. Dr. C. C. Iliescu”, Bucharest, Romania

**Keywords:** aortic stenosis, TAVI, coupling, right ventricle, pulmonary hypertension

## Abstract

**Introduction:**

Right ventricular (RV) dysfunction and pulmonary hypertension (PH) have been previously associated with unfavorable outcomes in patients with severe aortic stenosis (AS) undergoing transcatheter aortic valve implantation (TAVI), but little is known about the effect of right ventricle (RV) to pulmonary artery (PA) coupling. Our study aimed to evaluate the determinant factors and the prognostic value of RV-PA coupling in patients undergoing TAVI.

**Methods:**

One hundred sixty consecutive patients with severe AS were prospectively enrolled, between September 2018 and May 2020. They underwent a comprehensive echocardiogram before and 30 days after TAVI, including speckle tracking echocardiography (STE) for myocardial deformation analysis of the left ventricle (LV), left atrium (LA), and RV function. Complete data on myocardial deformation was available in 132 patients (76.6 ± 7.5 years, 52.5% men) who formed the final study population. The ratio of RV free wall longitudinal strain (RV-FWLS) to PA systolic pressure (PASP) was used as an estimate of RV-PA coupling. Patients were analyzed according to baseline RV-FWLS/PASP cut-off point, determined through time-dependent ROC curve analysis, as follows: normal RV-PA coupling group (RV-FWLS/PASP ≥0.63, *n* = 65) and impaired RV-PA coupling group (RV-FWLS/PASP < 0.63, *n* = 67).

**Results:**

A significant improvement of RV-PA coupling was observed early after TAVI (0.75 ± 0.3 vs. 0.64 ± 0.3 before TAVI, *p* < 0.001), mainly due to PASP decrease (*p* < 0.001). LA global longitudinal strain (LA-GLS) is an independent predictor of RV-PA coupling impairment before and after TAVI (OR = 0.837, *p* < 0.001, OR = 0.848, *p* < 0.001, respectively), while RV diameter is an independent predictor of persistent RV-PA coupling impairment after TAVI (OR = 1.174, *p* = 0.002). Impaired RV-PA coupling was associated with a worse survival rate (66.3% vs. 94.9%, *p*-value < 0.001) and emerged as an independent predictor of mortality (HR = 5.97, CI = 1.44–24.8, *p* = 0.014) and of the composite endpoint of death and rehospitalization (HR = 4.14, CI = 1.37–12.5, *p* = 0.012).

**Conclusion:**

Our results confirm that relief of aortic valve obstruction has beneficial effects on the baseline RV-PA coupling, and they occur early after TAVI. Despite significant improvement in LV, LA, and RV function after TAVI, RV-PA coupling remains impaired in some patients, it is mainly related to persistent pulmonary hypertension and is associated with adverse outcomes.

## Introduction

The treatment of severe aortic stenosis (AS) is guided by evidence-based recommendations (1–3). Transcatheter aortic valve implantation (TAVI) has become the preferred treatment of severe symptomatic AS in patients who are at high risk for surgery, with continuous expansion towards use in intermediate and low-risk patients (4–7). Although providing excellent short-term results, post-TAVI-associated 1–5 year mortality varies widely between 8.3% and 67.8% according to patients' risk profiles (5–7). This calls for a detailed examination of patient-related factors that have an impact on long-term survival (8). Among these, right ventricular (RV) dysfunction and pulmonary hypertension have been shown to have a negative prognostic impact after TAVI (9–11). Only a few studies have analyzed the synergic impact of these factors on mortality, by evaluating the RV to pulmonary artery (RV-PA) coupling, which integrates the RV systolic performance at a given degree of afterload through a non-invasive parameter, but using different parameters or in chronic heart failure patients (12, 13). Our study aims to assess the determinant factors and the prognostic value of RV-PA coupling, by using the ratio between right ventricle free wall longitudinal strain (RV-FWLS) and pulmonary artery systolic pressure (PASP), in patients undergoing TAVI.

## Methods

### Study population and procedure

Patients with severe symptomatic AS scheduled to undergo transfemoral TAVI in our center were prospectively enrolled between September 2018 and May 2020. Selection criteria included: age >40, severe AS [aortic valve area (AVA) < 1.0 cm^2^, indexed AVA < 0.6 cm^2^/m^2^, peak aortic jet velocity ≥4 m/s, or mean gradient ≥40 mmHg]. Exclusion criteria included: hypertrophic cardiomyopathy, prosthetic aortic valve, non-transfemoral TAVI, and poor acoustic window. All patients underwent Heart Team evaluation and were deemed eligible for TAVI based on current guideline recommendations. All procedures were performed in a hybrid operating room with participation of both interventional cardiologist and cardiovascular surgeon.

All patients underwent percutaneous transfemoral TAVI with balloon-expandable valve. The procedure was performed under general anesthesia and invasive hemodynamic monitoring. Transesophageal echocardiography was used during the procedure for additional guidance and assessment. Clinical, biological, and procedural data were collected. Coronary artery disease (CAD) was defined as the presence of coronary artery lesions, previously treated (PCI or CABG) or not requiring treatment at the time of procedure. The primary outcome was a composite endpoint of major adverse cardiac events (MACE) consisting of cardiac-related rehospitalization (obtained through a search in our institutional database and a telephone questionnaire), and all-cause mortality (obtained through a query of the National Register of population records), both performed 3 years after TAVI. No event was registered in the interval between intervention and follow-up echocardiography. The study was reviewed and approved by the institutional Ethics Committee.

### Definitions and data collection—echocardiographic evaluation

All patients underwent a comprehensive echocardiogram performed by experienced echocardiographers both before and 30 days after TAVI using a Vivid E95 ultrasound system (General Electric Healthcare, Horten, Norway). Data were digitally stored for offline analysis using commercially available software (EchoPac version 203; GE Medical Systems, Horten, Norway) and images were analyzed by a single trained cardiologist according to current guidelines (14). Evaluation included standard parameters used to assess AS severity: peak aortic jet velocity, peak and mean pressure gradients across the aortic valve (using modified Bernoulli equation), and AVA (using continuity equation). In the parasternal long-axis view, LV dimensions were assessed, and LV mass was calculated using Devereux's formula and indexed to body surface area (14). LV end-diastolic and end-systolic volumes were measured in the apical 4-chamber and 2-chamber views and indexed to body surface area (14). LV ejection fraction (EF) was calculated according to the Simpson's biplane method (14). Left atrial volumes were measured by the biplane method of disks and indexed for body surface area (14). Transmitral flow was assessed by PW Doppler to measure the peak early (E) and late (A) diastolic velocities, and tissue Doppler imaging of the mitral annulus on the apical 4- chamber view was used to measure the e' velocities at both the lateral and septal sites to calculate the E/e’ ratio.

Myocardial deformation analysis using speckle tracking echocardiography (STE) was performed to assess LV and LA function. Evaluation included STE analysis for LV function: LV global longitudinal strain (GLS); LA function: LA global longitudinal strain (LA*ε*, reservoir function), LA systolic strain rate (SSr, reservoir function), LA early diastolic strain rate (ESr, conduit function), late diastolic LA strain rate (ASr, contractile function). Complete myocardial deformation analysis was possible in 132 out of 160 patients. Negative values of strain parameters are used as moduli (positive numbers) for ease of analysis. RV function was assessed by measuring TAPSE, the peak systolic myocardial velocity at the lateral site of the tricuspid annulus (S'RV), RV fractional area change (FAC) and RV longitudinal strain parameters by STE: peak values of global RV strain (RV-GLS), RV free wall longitudinal strain (RV-FWLS) and the interventricular septum longitudinal strain (RV-IVS). The right ventricular systolic pressure was calculated from the peak velocity of the tricuspid regurgitant jet using the Bernoulli equation and the right atrial pressure (determined by the diameter and inspiratory collapse of the inferior vena cava) was added (14). Mean pulmonary arterial pressure was derived from pulmonary arterial systolic pressure (PASP) (15). The ratio of RV-FWLS to PASP was used as an estimate of RV-PA coupling. Patients were divided according to baseline RV-FWLS/PASP ratio as follows: RV-FWLS/PASP ≥ 0.63 as normal RV-PA coupling group (*n* = 65) and RV-FWLS/PASP < 0.63 as impaired RV-PA coupling group (*n* = 67) and were analyzed accordingly.

### Statistical analysis

Continuous variables are given as mean ± standard deviation and compared using the Student *t*-test. Discrete variables were expressed as counts and percentages, and comparisons between groups were done with the *χ*^2^ or Fisher`s exact test, when appropriate. For comparisons between subgroups, Kruskal–Wallis test, Wilcoxon rank sum tests using pairwise comparisons and Chi-square test for comparing proportions (of categorical variables) between >2 groups have been used. Bonferroni method was used to adjust *p*-values for multiple comparisons.

Long-term clinical outcomes were estimated using the Kaplan-Meier method, with comparisons made using the log-rank test (overall or pair wise as appropriate).

Univariable analysis (linear and binary logistic) was used to identify potential predictors of RV-PA coupling from baseline characteristics. After careful selection of variables based on clinical judgment, univariable assessment (*p* < 0.05), exclusion of variables showing collinearity (Pearson's coefficient >0.6), and multiple testing to ensure stability, a multivariable model has been fitted (by stepwise multivariable regression analysis, linear and binary logistic).

**Figure 1:**
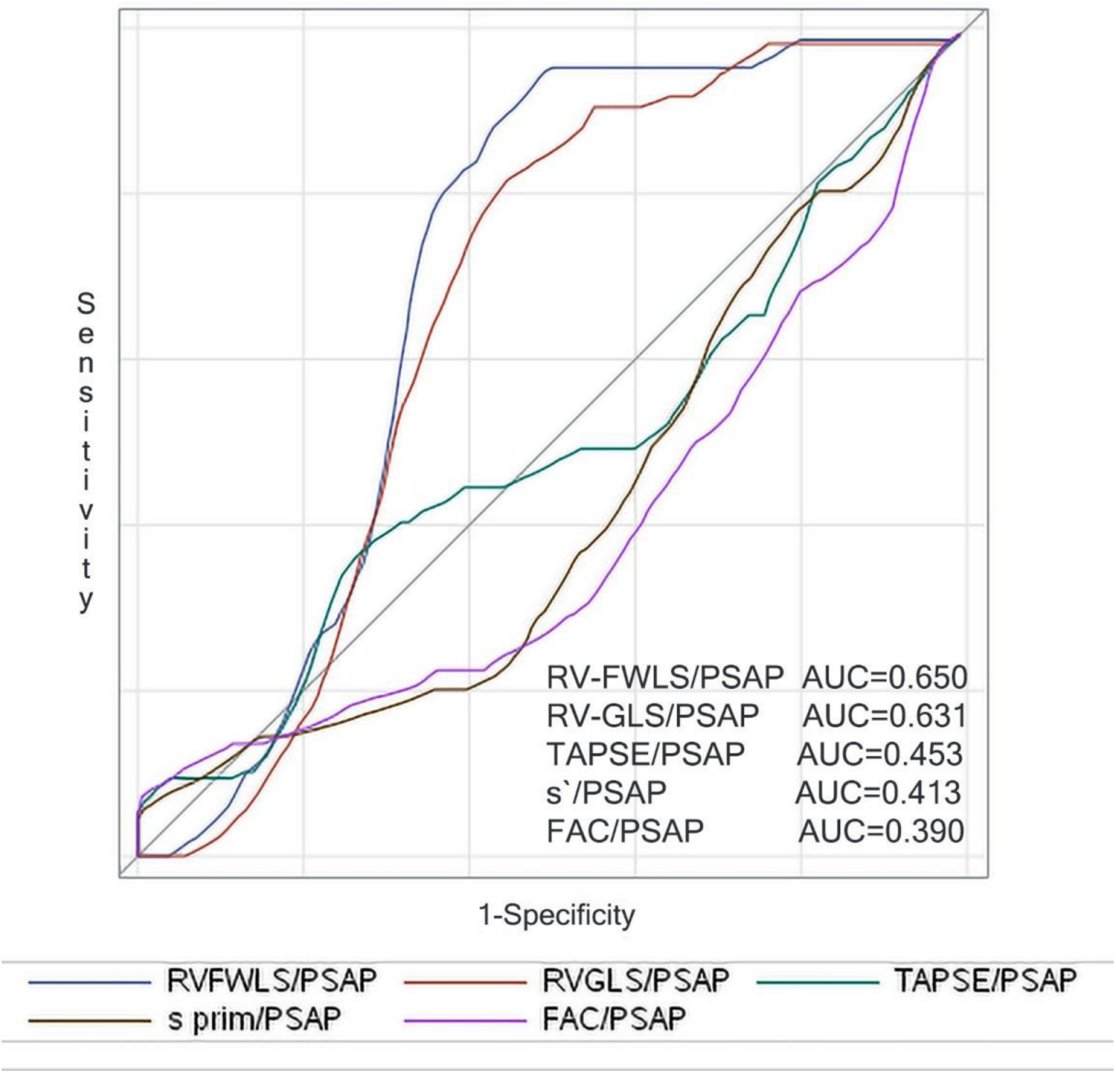
Right censored ROC curves for 3-year mortality of the parameters reflecting surrogates of baseline RV-PA coupling.

Univariable predictors of all-cause mortality were determined using Cox proportional hazards (Enter). Multivariable analysis was also performed in a similar fashion (Forward Wald). A two-sided *p*-value of 0.05 was considered statistically significant for all tests. Time-dependent receiver operating characteristic (ROC) analysis was used to determine the associations between individual and combined surrogate parameters of RV-PA coupling and 3-year mortality (Figure 1). The baseline RV-FWLS/PASP cut-off point of 0.63 to discriminate between normal and impaired RV-PA coupling was determined through time-dependent ROC curve analysis based on the highest sum of sensitivity and specificity (death—AUC 0.650, CI 0.60–0.70, *p* = 0.001; sensitivity 86%, specificity 57%;) and is within the same range of previous studies associated with survival in AS or heart failure patients (12, 16, 17). Time-dependent ROC curve analysis was performed using SAS, version 9.3, software (Cary, NC). The rest of the analyses were conducted using SPSS 21.0 (SPSS, Inc., Chicago, IL, USA).

## Results

### Baseline characteristics

Patients with impaired baseline RV-PA coupling were younger, had more often atrial fibrillation, prior myocardial infarction, angina, and higher NYHA functional class compared to patients with normal RV-PA coupling (Table 1). There were no differences between groups regarding comorbidities. Even though there were no significant differences between groups regarding peak aortic jet velocity and mean transvalvular gradient (Table 2), there was a higher incidence of bicuspid valve and smaller AVAi in the group with impaired RV-PA coupling as compared to patients with normal RV-PA coupling (*p* = 0.031, and *p* = 0.008, respectively). Impaired LVEF was more frequent, and the impairment was more severe in patients with impaired RV-PA coupling (*p* < 0.001). Additionally, patients with impaired RV-PA coupling had more advanced cardiac damage as suggested by larger LA dimension and lower LA-GLS, larger RA and RV dimensions, and lower parameters of RV function compared to normal RV-PA coupling group (*p* < 0.001).

**Table 1:** Clinical characteristics.

Demographic and clinical characteristics	All patients (*n* = 132)	Normal baseline RV-PA coupling	Impaired baseline RV-PA coupling	*p*-value
		RVFWLS/PASP ≥0.63 (*n* = 65)	RVFWLS/PASP <0.63 (*n* = 67)	
Age (years)	76.6 ± 7.5	78.0 ± 5.7	75.1 ± 8.6	0.024
Gender (female)	76 (47.5%)	30 (46.2%)	37 (55.2%)	0.297
BMI (kg/m^2^)	27.2 ± 4.5	26.8 ± 4.4	27.5 ± 4.6	0.375
**Symptoms**				
Angina	78 (59.1%)	44 (67.7%)	34 (50.7%)	0.048
NYHA functional class	2.7 ± 0.6	2.6 ± 0.6	2.7 ± 0.6	0.191
Class 2	39 (29.5%)	26 (40.0%)	13 (19.4%)	0.035
Class 3	86 (65.2%)	36 (55.4%)	50 (74.6%)	
Class 4	7 (5.3%)	3 (4.6%)	4 (6.0%)	
**Comorbidities**				
Atrial fibrillation	41 (31.1%)	11 (16.9%)	30 (44.8%)	0.001
CAD	74 (56.1%)	40 (61.5%)	34 (50.7%)	0.212
Prior PCI	35 (26.5%)	15 (23.1%)	20 (29.9%)	0.378
Obesity	45 (34.1%)	20 (30.8%)	25 (37.3%)	0.428
Type 2 DM	38 (29.0%)	21 (32.3%)	17 (25.8%)	0.402
COPD	13 (9.8%)	5 (7.7%)	9 (13.4%)	0.284
Anemia	57 (43.2%)	23 (35.4%)	34 (50.7%)	0.075
CKD (≥3)	31 (23.5%)	16 (24.6%)	15 (22.4%)	0.763
Frailty	54 (40.9%)	28 (43.1%)	26 (38.8%)	0.618

BMI, body mass index; DM, diabetes mellitus; CAD, coronary artery disease; CKD, chronic kidney disease; COPD, chronic obstructive pulmonary disease; NYHA, New York Hear Association Class; PCI, percutaneous coronary intervention.

Values are mean ± SD, *n* (%), or median (interquartile range).

**Table 2:** Echocardiographic characteristics.

Echocardiographic characteristics	All patients (*n* = 132)	Normal baseline RV-PA coupling	Impaired baseline RV-PA coupling	*p*-value
		RVFWLS/PASP ≥0.63 (*n* = 65)	RVFWLS/PASP <0.63 (*n* = 67)	
**Aortic stenosis severity**				
Vmax, m/s	4.67 ± 0.8	4.8 ± 0.8	4.6 ± 0.8	0.106
Mean gradient, mmHg	57.6 ± 19.3	59.7 ± 19.4	55.6 ± 19.3	0.233
AVAi, cm^2^/m^2^	0.4 ± 0.2	0.44 ± 0.2	0.37 ± 0.1	0.008
Bicuspid valve	19 (14.4%)	5 (7.7%)	14 (20.9%)	0.031
Aortic regurgitation	1.2 ± 0.7	1.3 ± 0.7	1.2 ± 0.7	0.742
**Left ventricle**				
LVEF, %	51.8 ± 12.2	55.0 ± 7.9	48.6 ± 14.9	0.001
LV-GLS, %	−12.4 ± 4.3	−14.2 ± 3.9	−10.5 ± 3.7	<0.001
LVMi, g/m^2^	180.3 ± 50.9	174 ± 47	187 ± 53	0.154
E/A	1.2 ± 0.8	0.84 ± 0.4	1.8 ± 0.9	<0.001
Mitral regurgitation	1.3 ± 0.6	1.28 ± 0.6	1.27 ± 0.6	0.934
**Left atrium**				
LAAi, cm^2^/m^2^	15.2 ± 3.3	14.2 ± 2.4	16.3 ± 3.6	<0.001
LAVi, ml/m^2^	55.2 ± 19.0	49.3 ± 13.4	61.3 ± 21.6	<0.001
LA-GLS, % (LA_ɛ_)	12.4 ± 6.9	16.1 ± 5.9	8.6 ± 5.6	<0.001
**Right ventricle**				
TAPSE, cm	2.0 ± 0.4	2.2 ± 0.4	1.9 ± 0.4	<0.001
S`RV, cm/s	10.4 ± 2.8	11.5 ± 2.5	9.4 ± 2.6	<0.001
FAC, %	0.59 ± 0.3	42.6 ± 6.5	38.9 ± 9.1	0.009
PASP, mmHg	40.5 ± 15	30.9 ± 8.4	49.2 ± 15.1	<0.001
PAPm, mmHg	26.3 ± 9.2	20.9 ± 5.2	32.0 ± 9.2	<0.001
RA, mm	0.41 ± 0.1	33.8 ± 6.7	41.4 ± 8.5	<0.001
RV, mm	37.7 ± 8.4	32.0 ± 4.7	36.6 ± 6.4	<0.001
Tricuspid regurgitation	0.98 ± 0.87	0.85 ± 0.83	1.2 ± 0.90	0.014
RV-GLS, %	34.3 ± 6.1	−21.2 ± 4.3	−13.6 ± 4.9	<0.001
RV-FWLS, %	−17.4 ± 6.0	−27.0 ± 4.9	−16.9 ± 6.1	<0.001
RV-IVS, %	−22.0 ± 7.5	−13.4 ± 6.1	−8.6 ± 5.7	<0.001

Values are mean ± SD, *n* (%), or median (interquartile range). Vmax, maximum aortic velocity; AVA, aortic valve area; AVAi, aortic valve area index; LVEF, left ventricle ejection fraction; LV-GLS, left ventricle global longitudinal strain; LA, left atrium; LAAi, LA area index; LAVi, LA volume index; LVMi, LV mass index; LA-GLS, LA global longitudinal strain; TAPSE, tricuspid annular plane systolic excursion; S`RV, peak systolic myocardial velocity at the lateral site of the tricuspid annulus; PASP, systolic pulmonary artery pressure; PAPm, mean pulmonary artery pressure; FAC, right ventricle fractional area change; RA, right atrium; RV, right ventricle; RV-GLS, RV global longitudinal strain; RV-FWLS, RV free wall longitudinal strain; RV-IVS, RV interventricular septum strain.

### Echocardiographic changes after TAVI

All echocardiographic parameters describing AS severity improved significantly after the procedure (Table 3). Compared with baseline, there was a significant improvement of LVEF after TAVI (*p* = 0.008) and decrease of LV mass index (*p* < 0.001). Additionally, mitral valve regurgitation decreased after TAVI (*p* = 0.003) and there was also a decrease of LA volume (*p* = 0.007), and an improvement of LA function (*p* < 0.001). We found a significant improvement in RV-PA coupling after TAVI (*p* = 0.007), mainly driven by a decrease in PASP (*p* < 0.001).

**Table 3:** Echocardiographic changes after TAVI.

All patients (*n* = 132)	Baseline	post-TAVI	*p*-value
**Aortic stenosis severity**			
Vmax, m/s	4.67 ± 0.8	2.2 ± 0.5	<0.001
Mean gradient, mmHg	57.6 ± 19.4	12.1 ± 5.0	<0.001
AVA, cm^2^	0.72 ± 0.3	1.7 ± 0.6	<0.001
AVAi, cm^2^/m^2^	0.4 ± 0.2	1.0 ± 0.3	<0.001
Aortic regurgitation	1.2 ± 0.7	0.8 ± 0.6	<0.001
**Left ventricle**			
LVEF, %	51.4 ± 12.1	55.1 ± 10.0	0.008
LV-GLS, %	−12.3 ± 4.2	−13.9 ± 4.0	0.002
LVMi, g/m^2^	180.8 ± 50.5	155.8 ± 42.7	<0.001
E/A	1.2 ± 0.8	0.9 ± 0.4	0.001
Mitral regurgitation	1.3 ± 0.6	1.1 ± 0.5	0.003
**Left atrium**			
LAAi, cm^2^/m^2^	15.3 ± 3.2	14.2 ± 3.2	0.007
LAVi, ml/m^2^	55.3 ± 19.0	49.1 ± 18.2	0.007
LA-GLS, % (LA_ɛ_)	12.4 ± 6.9	15.6 ± 7.3	<0.001
**Right ventricle**			
TAPSE, cm/s	2.0 ± 0.4	2.1 ± 0.4	0.374
S’RV, cm/s	10.4 ± 2.8	12.3 ± 6.8	0.004
FAC, %	0.41 ± 0.1	0.42 ± 0.1	0.017
PASP, mmHg	40.2 ± 15	33.7 ± 11	<0.001
PAPm, mmHg	26.3 ± 9	22.1 ± 7	<0.001
RA, mm	37.7 ± 8.4	36.3 ± 7.9	0.178
RV, mm	34.3 ± 6.1	33.7 ± 5.9	0.378
Tricuspid regurgitation	0.98 ± 0.86	0.97 ± 0.77	0.944
RV-GLS, %	−17.3 ± 6.0	−18.6 ± 5.7	0.083
RV-FWLS, %	−21.9 ± 7.5	−23.2 ± 7.7	0.168
RV-IVS, %	−11.1 ± 6.2	−13.0 ± 5.1	0.010
TAPSE/PASP, cm/mmHg	0.59 ± 0.3	0.71 ± 0.3	<0.001
RV-GLS/PSAP	0.51 ± 0.27	0.61 ± 0.26	0.002
RV-FWLS/PSAP	0.64 ± 0.34	0.75 ± 0.33	0.007

Values are mean ± SD. TAVI, transcatheter aortic valve implantation. Vmax, maximum aortic velocity; AVA, aortic valve area; AVAi, aortic valve area index; LVEF, left ventricle ejection fraction; LV-GLS, left ventricle global longitudinal strain; LA, left atrium; LAAi, LA area index; LAVi, LA volume index; LVMi, LV mass index; LA-GLS, LA global longitudinal strain; TAPSE, tricuspid annular plane systolic excursion; S`RV, peak systolic myocardial velocity at the lateral site of the tricuspid annulus; PASP, systolic pulmonary artery pressure; PAPm, mean pulmonary artery pressure; FAC, right ventricle fractional area change; RA, right atrium; RV, right ventricle; RV-GLS, RV global longitudinal strain; RV-FWLS, RV free wall longitudinal strain; RV-IVS, RV interventricular septum strain.

Significant improvement of echocardiographic parameters describing AS severity after TAVI were further noted regardless of group (Table 4). LV remodeling and LV function improvement were significant after the procedure regardless of baseline RV-PA coupling status. LA function and volume significantly improved after TAVI in both groups. RA diameter significantly decreased after TAVI in the impaired RV-PA coupling group (*p* = 0.046). RV function improved after TAVI in the impaired RV-PA coupling group as measured by RV-GLS (*p* = 0.001), RV-FWLS (*p* = 0.003), RV-IVS (*p* = 0.003), S`RV (*p* = 0.026), but not by TAPSE (*p* = 0.187) and FAC (*p* = 0.060).

**Table 4:** Echocardiographic changes after TAVI according to baseline RV-PA coupling impairment (short-term effect of TAVI on RV-PA coupling).

Echocardiographic parameters	Normal RV-PA coupling baseline RVFWLS/PASP ≥0.63 (*n* = 65)			Impaired RV-PA coupling baseline RVFWLS/PASP <0.63 (*n* = 67)		
	Baseline	1 m post-TAVI	*p*-value	Baseline	1 m post TAVI	*p*-value
**Aortic stenosis severity**						
Vmax, m/s	4.8 ± 0.8	2.3 ± 0.5	<0.001	4.6 ± 0.8	2.2 ± 0.5	<0.001
AVAi, cm^2^/m^2^	0.44 ± 0.2	0.98 ± 0.4	<0.001	0.37 ± 0.1	0.92 ± 0.3	<0.001
Aortic regurgitation	1.3 ± 0.7	0.9 ± 0.6	<0.001	1.2 ± 0.7	0.8 ± 0.6	<0.001
**Left ventricle**						
LVEF, %	55.0 ± 7.9	57.5 ± 6.3	0.041	48.0 ± 14.4	52.8 ± 12.4	0.042
LV-GLS, %	−14.2 ± 3.9	−15.3 ± 3.6	0.098	−10.5 ± 3.7	−12.6 ± 3.9	0.002
LVMi, g/m^2^	174 ± 47	151 ± 41	0.003	187 ± 53	160 ± 44	0.002
E/A	0.84 ± 0.4	0.75 ± 0.3	0.167	1.8 ± 0.9	1.1 ± 0.6	<0.001
Mitral regurgitation	1.28 ± 0.6	1.05 ± 0.4	0.007	1.27 ± 0.6	1.12 ± 0.5	0.111
**Left atrium**						
LAAi, cm^2^/m^2^	14.2 ± 2.4	13.3 ± 2.5	0.047	16.3 ± 3.6	15.0 ± 3.7	0.032
LAVi, ml/m^2^	49.3 ± 13.4	44.5 ± 12.9	0.042	61.3 ± 21.6	53.5 ± 21.3	0.037
LA-GLS, % (LA_ɛ_)	16.1 ± 5.9	19.1 ± 6.3	0.007	8.6 ± 5.6	12.1 ± 6.5	0.001
**Right ventricle**						
TAPSE, cm/s	2.2 ± 0.4	2.2 ± 0.4	0.936	1.9 ± 0.4	2.0 ± 0.4	0.187
S`RV, cm/s	11.5 ± 2.5	12.4 ± 2.2	0.020	9.4 ± 2.6	12.1 ± 9.3	0.026
FAC, %	42.6 ± 6.5	44.6 ± 6.7	0.084	38.9 ± 9.1	41.7 ± 8.2	0.060
PASP, mmHg	30.9 ± 8.4	29.1 ± 7.0	0.191	49.2 ± 15.1	38.1 ± 13.1	<0.001
PAPm	20.9 ± 5.2	19.8 ± 4.3	0.191	32.0 ± 9.1	25.3 ± 8.0	<0.001
RV-GLS, %	−21.2 ± 4.3	−20.9 ± 5.1	0.732	−13.6 ± 4.9	−16.5 ± 5.5	0.001
RV-FWLS, %	−27.0 ± 4.9	−26.1 ± 6.7	0.424	−16.9 ± 6.1	−20.6 ± 7.5	0.003
RV-IVS, %	−13.4 ± 6.1	−14.6 ± 4.5	0.244	−8.6 ± 5.2	−11.5 ± 5.1	0.003

Values are mean ± SD, *n* (%), or median (interquartile range). Vmax, maximum aortic velocity; AVA, aortic valve area; AVAi, aortic valve area index; LVEF, left ventricle ejection fraction; LV-GLS, left ventricle global longitudinal strain; LA, left atrium; LAAi, LA area index; LAVi, LA volume index; LVMi, LV mass index; LA-GLS, LA global longitudinal strain; TAPSE, tricuspid annular plane systolic excursion; S`RV, peak systolic myocardial velocity at the lateral site of the tricuspid annulus; PASP, systolic pulmonary artery pressure; PAPm, mean pulmonary artery pressure; FAC, right ventricle fractional area change; RA, right atrium; RV, right ventricle; RV-GLS, RV global longitudinal strain; RV-FWLS, RV free wall longitudinal strain; RV-IVS, RV interventricular septum strain.

### Predictors of impaired RV-PA coupling

RV-PA coupling correlates at univariable and multivariable logistic regression analysis are presented in Table 5. Before TAVI, LA-GLS and RA diameter were independent predictors of RV-PA coupling impairment. Age, LA-GLS and RV diameter emerged as independent predictors of impaired RV-PA coupling after TAVI.

**Table 5:** Univariable and multivariable predictors of RV-PA coupling impairment (binary logistic regression).

Univariable regression analysis	Pre TAVI		Post TAVI	
	OR	*p*-value	OR	*p*-value
Age	0.940	0.028	0.952	0.044
Atrial fibrillation	3.980	0.001	9.389	<0.001
AVAi	0.048	0.009	0.406	0.102
Bicuspid	3.170	0.001	3.786	0.999
LVMi	1.005	0.129	1.009	0.043
LVEF	0.947	<0.001	0.951	0.008
LV-GLS*	1.305	<0.001	1.290	<0.001
E/A*	14.46	<0.001	4.689	0.020
LAVi	1.051	<0.001	1.046	0.001
LAAi	1.315	<0.001	1.251	0.001
LA-GLS	0.803	<0.001	0.845	<0.001
TAPSE	0.820	<0.001	0.900	0.030
S’RV*	0.715	<0.001	1.003	0.902
FAC*	0.942	0.011	0.897	<0.001
PASP*	1.156	<0.001	1.140	<0.001
PAPm*	1.267	<0.001	1.239	<0.001
RA	1.149	<0.001	1.107	<0.001
RV	1.179	<0.001	1.139	<0.001
Tricuspid regurgitation	1.687	0.017	1.750	0.024
RV-GLS*	1.483	<0.001	1.480	<0.001
RV-FWLS*	1.434	<0.001	1.361	<0.001
RV-IVS*	1.181	<0.001	1.227	<0.001
**Multivariable regression analysis—Model 1 (Pre TAVI)**				
LA-GLS	0.837	<0.001		
RA	1.111	0.003		
**Multivariable regression analysis—Model 2 (Post TAVI)**				
Age			0.931	0.040
LA-GLS			0.848	<0.001
RV			1.174	0.002

AVA, aortic valve area; AVAi, aortic valve area index; LVEF, left ventricle ejection fraction; LV-GLS, left ventricle global longitudinal strain; LA, left atrium; LAAi, LA area index; LAVi, LA volume index; LVMi, LV mass index; LA-GLS, LA global longitudinal strain; TAPSE, tricuspid annular plane systolic excursion; S`RV, peak systolic myocardial velocity at the lateral site of the tricuspid annulus; PAPS, systolic pulmonary artery pressure; PAPm, mean pulmonary artery pressure; FAC, right ventricle fractional area change; RA-right atrium; RV, right ventricle RV-GLS, RV global longitudinal strain; RV-FWLS, RV free wall longitudinal strain; RV-IVS, RV interventricular septum strain. Model 1—Multivariable analysis (Backward Wald)—variables: age, atrial fibrillation, AVAi, bicuspid, LAVi, LA-GLS, LVEF, RA, RV, tricuspid regurgitation. Model 2—Multivariable analysis (Backward Wald)—variables: age, atrial fibrillation, LAVi, LV mass index, LA-GLS, LVEF, RA, RV, tricuspid regurgitation.

*Variables highly correlated with another variable (Pearson coefficient > 0.6), not included.

### Clinical outcomes

**Figure 2:**
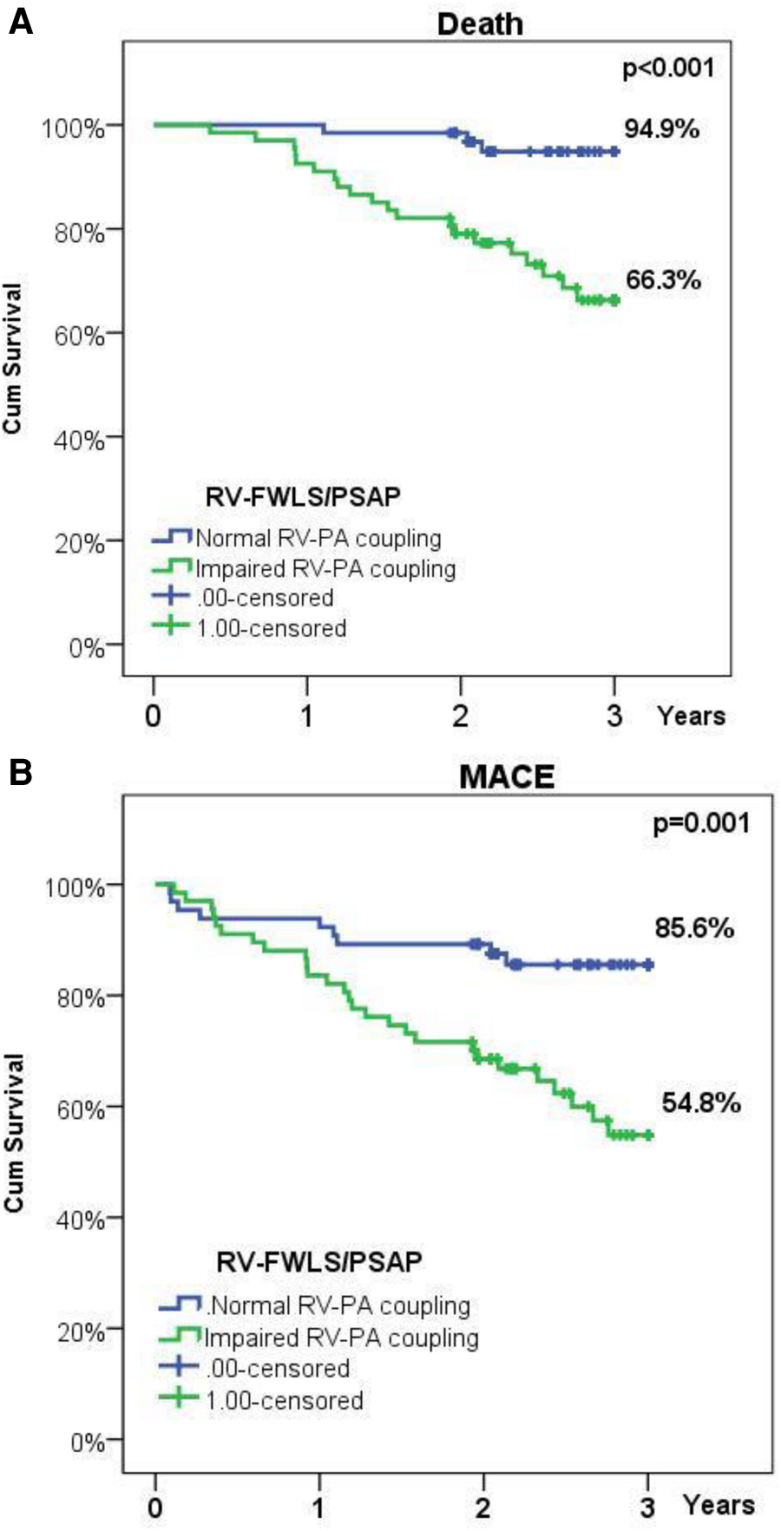
(**A**) Kaplan-Meier survival plot illustrating survival in patients with normal or impaired baseline RV-PA coupling; (**B**) Kaplan-Meier survival plot illustrating freedom from rehospitalization or death in patients with normal or impaired baseline RV-PA coupling.

Follow-up data were available for all patients, mean follow-up lasting 2.47 years (903 ± 216 days, range: 134–1,095 days). During follow-up, MACE occurred in 38 patients (24.4%), of which rehospitalization in 19 patients (11.9%), and death in 25 patients (15.6%). At 3-year follow-up the survival rate was 82.1%. Kaplan Meier analysis revealed that impaired baseline RV-PA coupling was associated with worse outcomes: lower freedom from MACE (54.8% vs. 85.6% in normal RV-PA coupling, *p*-value = 0.001) and lower survival rate (66.3% vs. 94.9% in normal RV-PA coupling, *p*-value < 0.001) (Figure 2). Impaired baseline RV-PA coupling as quantified by RV-FWLS/PSAP emerged as an independent predictor of both mortality (HR = 5.97, CI = 1.44–24.8, *p*-value = 0.014) and MACE (HR = 4.14, CI = 1.37–12.5, *p*-value = 0.012) (Table 6).

**Table 6:** Predictors of MACE and mortality (Cox regression analysis).

Univariable analysis	MACE			Death		
Baseline parameters	HR	95% CI	*p*-value	HR	95% CI	*p*-value
Atrial fibrillation	2.18	1.14–4.16	0.018	2.27	1.02–5.07	0.044
LAVi	1.01	0.99–1.02	0.110	1.02	0.99–1.03	0.059
LAVi > 44*	2.40	1.01–5.73	0.049	3.11	0.94–10.4	0.065
LA-GLS	0.96	0.91–1.01	0.110	0.96	0.90–1.02	0.160
LA-GLS > 14*	0.42	0.19–0.91	0.027	0.31	0.11–0.91	0.032
LAAi	1.07	0.99–1.16	0.097	1.10	1.00–1.21	0.047
LAAi > 14*	2.67	1.23–5.82	0.013	2.67	1.20–10.2	0.021
PASP	1.03	1.01–1.05	0.007	1.03	1.00–1.05	0.030
PASP > 60*	3.06	1.39–6.71	0.005	3.63	1.44–9.15	0.006
TAPSE/PASP	0.26	0.66–1.06	0.061	0.50	0.10–2.55	0.401
TAPSE/PASP < 0.36*	3.12	1.64–5.92	0.001	2.41	1.07–5.42	0.034
RV-FWLS	1.03	0.99–1.08	0.128	1.05	0.99–1.10	0.082
RV-FWLS/PASP	3.77	1.25–11.4	0.019	5.51	1.32–23.0	0.019
RV-FWLS/PASP < 0.63*	3.27	1.54–6.97	0.002	7.39	2.19–24.9	0.001
Multivariable analysis	MACE			Death		
RV-FWLS/PASP	4.14	1.37–12.5	0.012	5.97	1.44–24.8	0.014

MACE, major adverse cardiac events; LAVi, LA volume index; LAAI, LA area index; LA-GLS, LA global longitudinal strain; TAPSE, tricuspid annular plane systolic excursion; PAPS, systolic pulmonary artery pressure; PAPm, mean pulmonary artery pressure; RV-FWLS, RV free wall longitudinal strain; RV-GLS, RV global longitudinal strain. Multivariable analysis (Backward Wald)—variables: atrial fibrillation, LAVi, LAAi, LA-GLS, RV-FWS/PSAP.

*Cut-off values determined by ROC analysis.

## Discussion

Our study provides evidence that baseline RV-FWLS/PASP ratio, as a non-invasive surrogate of RV-PA coupling in patients with severe AS undergoing TAVI, is a novel parameter that refines risk assessment and independently predicts outcomes. The main findings of our study are: 1. Baseline RV-PA coupling impairment is influenced by persistent pulmonary hypertension and is associated with a greater burden of cardiac damage; 2. cardiac damage is only partially reversible, despite significant improvement of LV, LA, and RV function after TAVI; 3. Baseline impaired RV-PA coupling improves early after the procedure but continues to present a higher mortality risk in the long term.

### RV-PA coupling and aortic stenosis severity

Although some studies reported no correlation between AS severity and the presence of PH or RV dysfunction, we found that patients with impaired baseline RV-PA coupling had more severe AS with lower AVAi and had more often bicuspid valves (13, 18, 19). The fact that there were no significant differences between groups regarding peak aortic jet velocity, peak and mean transvalvular gradients, should be interpreted in the context of impaired LVEF and low-flow low-gradient AS, which was more frequent in patients with impaired baseline RV-PA coupling. Moreover, AS severity traditionally quantified by transvalvular gradient and AVA has recently suffered a paradigm shift, resulting in the concept of AS-related cardiac damage or injury according to the reversibility potential (20, 21). As this model better translates AS severity into prognosis, in depth analysis of each component of cardiac damage/injury is required to improve treatment strategies and timing with respect to reversibility of injury (22, 23).

### RV-PA coupling and left ventricular function in aortic stenosis

Even though the impact of impaired LVEF on RV-PA coupling has not been extensively studied, it has been previously reported that impaired LVEF is an independent predictor of PH (24). Due to ventricular interdependence, a significant fraction of developed pressure and RV volume outflow depends on LVEF, resulting in frequent RV dysfunction in patients with AS and is associated with reduced survival (25). Our study population included patients with impaired LVEF, more than a quarter, with a higher prevalence in the group with impaired RV-PA coupling. While LVEF and LV-GLS predicted to some extent baseline RV-PA coupling impairment, they failed to be independent predictors in the multivariable analysis. This could suggest that ventricular interdependence plays only a secondary role in the equation of RV-PA coupling. In our study we observed that baseline RV-PA coupling correlated with LV diastolic dysfunction but failed to independently predict it, which could indicate that despite reversible LV injury, impairment of upstream cardiac components could have different reversibility.

### RV-PA coupling and left atrial function in aortic stenosis

Although LA function and dimensions have been previously shown to have an impact on morbidity and mortality in AS, little is known about the link between RV-PA coupling and LA function (26–28). In the context of AS, LA enlargement is a marker of longstanding increased LV filling pressures, and has been further correlated with upstream increased pressures in the pulmonary circulation (29).

In our study impaired baseline RV-PA coupling was associated with larger LA dimensions and impaired LA function, especially the reservoir and booster-pump. Atrial fibrillation was also correlated with impaired baseline RV-PA coupling, indicating loss of LA booster-pump. LA-GLS was associated with impaired RV-PA coupling before and after TAVI, suggesting that persistently impaired RV-PA coupling may be linked to irreversibility of atrial dysfunction. From a clinical point of view, improvement of left atrial function in the context of impaired RV-PA coupling failed to offer a significant benefit in terms of morbidity and mortality. While both indexed LA area and volume decreased after the procedure, persistence of LA dilation predicts the persistence of RV-PA coupling impairment. This clinically translated into the fact that the degree of decrease in LA dimensions only marginally impacted outcomes. These data are consistent with previous studies which suggest that LA active emptying is impaired in the presence of severe LA dilation, and propose the exceeding of optimal Frank-Starling mechanism as the explanation (30).

### RV-PA coupling in aortic stenosis

Baseline pulmonary hypertension (PH) is common in patients with AS undergoing TAVI and has been linked to increased morbidity and mortality (31–33). Although more controversial regarding evaluation, quantification and impact, baseline RV dysfunction is also associated with adverse outcomes (21, 34, 35). The connection between PH and RV function has complex underlying pathophysiologic mechanisms that can be partly expressed through RV-PA coupling (36). Several studies have shown that TAPSE/PASP ratio as a non-invasive surrogate of RV-PA coupling offers prognostic information in patients with severe AS (12, 13). While PH can improve after TAVI and is associated with improved survival, similar to patients without PH, persistent PH is strongly associated with increased mortality and may require further treatment (33). Our study indicates an improvement in RV-PA coupling after TAVI, mainly through decrease in PASP values. Although not reflected by all RV function parameters, early improvement of RV function was noted, as assessed by tricuspid lateral annulus systolic velocity, FAC, RV-GLS and RV-FWLS. Acute improvements in RV function after TAVI have been previously demonstrated and can be partly explained by the LV-RV systolic interaction (37, 38). A study on HFrEF patients, where both RVGLS and RV-FWS have prognostic value, has shown that RV-FWS better predicts outcome, mainly because it is less influenced by LV longitudinal dysfunction (39). The results of our study, together with previous findings, support the idea that baseline RV-FWLS better refines risk assessment when used as a surrogate parameter for RV function normalized to baseline PASP value in the RV-PA coupling equation.

### Limitations

This is a prospective study conducted on consecutive AS patients meeting the eligibility criteria for TAVI, resulting in a heterogenous population in terms of associated comorbidities, but resembling the real-life clinical setting. More than half of the included patients had associated CAD (of which half underwent previous coronary revascularization), as CAD is the most common comorbidity in AS (40).

One limitation of the study consisted in the short follow-up period as comprehensive echocardiographic evaluation was not routinely performed after the 1-month follow-up visit. Another limitation of the study is the lack of invasive measurements for comparison, but RV-FWLS/PASP has already been validated as a surrogate of RV-PA coupling as Ees/Ea in other populations (12, 41, 42) While mortality at 3-years was obtained through queries of the National Register of population records, no data regarding cause of death was available. Nonetheless, all-cause mortality represents an objective and relevant outcome. The pathophysiologic relations outlined in our study need to be interpreted in the context of a relatively small number of patients, but with comprehensive advanced echocardiographic assessment, and require further confirmation in larger studies.

## Conclusion

Our results confirm that relief of aortic valve obstruction by TAVI has beneficial effects on the RV-PA coupling, that occur early after the procedure. This is accompanied by a significant improvement in LV, LA, and RV function. The results show a significant correlation between LA function and RV-PA coupling before TAVI, suggesting the contribution of LA function in modulating right heart function in patients with AS. Persistence of impaired RV-PA coupling after TAVI is mainly influenced by persistent pulmonary hypertension and is associated with long-term adverse outcomes. The complex underlying mechanisms of RV-PA coupling impairment require further analysis of cardiac injury reversibility.

## Data Availability

Requests to access these datasets should be directed to bogdan.a.popescu@gmail.com.
